# Childhood Psychological Maltreatment, Psychological Flexibility, Family Conflict, and Subjective Happiness in University Students: A Serial Mediation Model

**DOI:** 10.1007/s11126-025-10134-w

**Published:** 2025-04-03

**Authors:** Muhammed Akat, Sinan Okur, Ömer Faruk Akbulut, Seydi Ahmet Satıcı, Erdal Hamarta

**Affiliations:** 1 Faculty of Education, Department of Psychological Counseling, Karamanoglu Mehmetbey University, Karaman, Türkiye; 2 Department of Educational Sciences, National Defense University, Turkish Air Force Academy, Istanbul, Türkiye; 3 Quality Coordinatorship Unit, Afyonkarahisar Health Sciences University, 03030 Afyonkarahisar, Türkiye; 4 Faculty of Education, Department of Psychological Counseling, Yildiz Technical University, Istanbul, Türkiye; 5 Ahmet Kelesoglu Faculty of Education, Department of Psychological Counseling, Necmettin Erbakan University, Konya, Türkiye

**Keywords:** Childhood psychological maltreatment, Psychological flexibility, Family conflict, Subjective happiness, Serial mediation analysis

## Abstract

Childhood psychological maltreatment has psychological, behavioral, and emotional repercussions on individuals in adulthood. These reflections play a role in both the internal distress of individuals and the deterioration of their interpersonal relationships. The aim of this study was to examine the serial mediating role of psychological flexibility and family conflict in the relationship between childhood psychological maltreatment and subjective happiness. The study was conducted with 493 university students (61.7% female, 38.3% male), with ages ranging from 18 to 53 years (*M*_*age*_ = 24.02, *SD* = 6.342). The data of this research was analyzed using structural equation modeling. The findings of the study indicate that psychological flexibility and family conflict have a serial mediating role in the relationship between childhood psychological maltreatment and subjective happiness. The results demonstrated that to improve the subjective happiness of people who have been psychologically abused as children, it is necessary to make them more psychologically flexible and teach them how to deal with family conflict. Overall, these findings emphasize the importance of fostering psychological flexibility and conflict resolution skills as key intervention targets to mitigate the long-term negative effects of childhood psychological maltreatment on subjective happiness. The findings of the study were discussed and interpreted in detail in the light of the relevant literature.

## Introduction

The pursuit of happiness is a natural consequence of being human. People's search for happiness has led scientists to conduct research on its causes and consequences. Happiness consists of cognitive and emotional evaluations that reveal people's satisfaction with their lives [70]. In other studies, happiness is defined as experiencing positive emotions more than negative emotions and being satisfied with life [23, 24]. Based on these definitions, greater positive emotions and satisfaction with life can increase happiness levels. Furthermore, there are some factors that reduce the happiness levels of individuals. One of these factors is exposure to childhood psychological maltreatment [33].

Childhood psychological maltreatment is a repetitive parental pattern that harms the child's development and/or makes the child feel unloved, unwanted, worthless, defective, or valued only when meeting the needs of others [29]. According to the definition of the American Professional Society on the Abuse of Children [5], childhood psychological maltreatment consists of acts of neglect and abuse such as spurning, terrorizing, exploiting/corrupting, denying emotional responsiveness, isolating, and mental, health, medical, and educational neglect. The widespread encounter with psychological maltreatment in the world influences these definitions. The World Health Organization [67] reports that approximately 60% of children under the age of five (about 400 million children) are exposed to physical punishment and/or psychological violence by their parents or caregivers. Additionally, 20% of women and 14% of men report experiencing sexual abuse during childhood. In the European Region, the prevalence rate of mental or emotional abuse was determined to be 29.1% [68]. The current report published by UNICEF [63] states that approximately 400 million children under the age of five are exposed to psychological attacks or physical punishment at home. A systematic study, examining ten studies covering 14,360 children from different continents of the world, found that the prevalence rate of psychological maltreatment during the COVID-19 period was 39% [43]. These statistics highlight the alarming prevalence of child maltreatment worldwide and emphasize the necessity of addressing its long-term psychological consequences.

Childhood psychological maltreatment is strongly associated with a wide range of developmental, educational, emotional, and behavioral problems [8, 12]. Individuals exposed to childhood psychological maltreatment face an increased risk of experiencing mental health issues in later years, including social anxiety, depression, suicidal ideation, and maladaptive emotion regulation strategies (e.g., [61, 64, 65, 75]). Beyond its psychological consequences, childhood psychological maltreatment has been linked to problematic technology use, including excessive internet gaming, smartphone use, and social media engagement, as well as substance use (e.g., [11, 45, 66, 69]). These findings suggest that childhood psychological maltreatment may increase vulnerability to addictive behaviors, possibly as a maladaptive coping mechanism for emotional distress.

Additionally, childhood psychological maltreatment has far-reaching social consequences. Individuals exposed to psychological maltreatment in childhood are more likely to develop maladaptive interpersonal patterns, such as increased aggression [4] and diminished positive family communication in adulthood [56]. Moreover, childhood psychological maltreatment negatively impacts subjective happiness by disrupting key psychological mechanisms. Childhood psychological maltreatment can lead to insecure attachment patterns [15], and maladaptive cognitive processes [74], all of which reduce an individual's ability to experience and sustain positive emotions. Additionally, it undermines fundamental psychological needs, such as autonomy and self-worth, which are essential for well-being [20]. These effects not only impact individual well-being but also contribute to broader societal challenges. In summary, existing research highlights the profound and multifaceted impact of childhood psychological maltreatment, underscoring the need for early interventions to mitigate its long-term psychological and social consequences.

Considering that mental health problems lead to a decrease in adults' happiness levels, it can be said that psychological maltreatment is associated with low happiness in adults. Indeed, a large body of recent empirical evidence suggests that psychological maltreatment can trigger fear of happiness and prevent positive emotions [10, 56]. Based on these studies, the importance of regulating and expressing the feeling of happiness becomes apparent. Among the basic developmental characteristics of childhood is the ability to regulate and express emotions. Parents play an important role in shaping their children's emotions by giving the message that some emotions are acceptable through the language they use in their communication with their children [42, 60]. Children who are maltreated by their parents may experience negative emotions more intensely and have difficulty regulating their emotions [73]. Indeed, a longitudinal study found that childhood maltreatment was associated with emotional lability‐negativity, which in turn led to poor emotion regulation [41]. As a result, psychological maltreatment may reduce individuals' happiness levels. In addition to all these, there may be other variables that may play a role in the relationship between childhood psychological maltreatment and subjective happiness. Considering that subjective happiness is influenced by individual and environmental factors [22], psychological flexibility (personal factor) and family conflict (environmental factor) were considered as mediating variables in this study.

### Psychological Flexibility and Family Conflict as Serial Mediators

Psychological flexibility plays a preventive role in reducing psychological distress and serves as a protective factor for mental well-being. There are different definitions of psychological flexibility in the literature. Hayes et al. [32], for instance, defined psychological flexibility as the ability to be in the present moment, free from past or future fixations, and to exhibit behaviors that align with one's values. Within the framework of Acceptance and Commitment Therapy (ACT), psychological flexibility is considered essential for adaptive functioning, as it enables individuals to respond flexibly to internal experiences rather than being rigidly controlled by them [30]. In another explanation, the emotion, thought, and behavior dimensions of psychological flexibility are emphasized. According to this conceptualization, psychological flexibility includes being aware of emotions and thoughts without being negatively affected by them and exhibiting, maintaining, or changing behaviors within the framework of values [30, 49].

Psychological flexibility consists of six components: defusion, acceptance, self-as-context, contact with the present moment, values, and committed action [52]. These components collectively facilitate psychological resilience, allowing individuals to engage meaningfully with life despite emotional distress [44]. Individuals who have this interconnected characteristic have high levels of psychological flexibility. On the other hand, there are six basic characteristics that distance individuals from psychological flexibility and are conceptualized as psychological rigidity. These characteristics include dominance of the conceptualized past and feared future, weak self-knowledge, cognitive fusion, experiential avoidance, attachment to the conceptualized self, lack of values and clarity, dominance of pliance and inaction, impulsivity, or avoidant tracking [30]. Exposure to childhood psychological maltreatment may impair psychological flexibility by reinforcing experiential avoidance and cognitive fusion, making it difficult for individuals to disengage from distressing past experiences [39]. Childhood psychological maltreatment may also limit their ability to act in accordance with personal values, thereby reducing committed action and overall psychological adaptability. These findings suggest that strengthening psychological flexibility, particularly through ACT-based interventions, may serve as an effective therapeutic approach for individuals exposed to childhood psychological maltreatment. All this theoretical information about psychological flexibility demonstrates that psychological flexibility supports the basic mechanisms underlying the individual's ability to adapt.

Psychological flexibility supports problem-solving and coping skills that enable individuals to effectively cope with changing environmental conditions and challenging life events [19, 55]. Simultaneously, studies have demonstrated that psychological flexibility supports psychological resources such as psychological resilience, life satisfaction, positive affect, eudaimonic wellbeing, relationship quality, and self-compassion (e.g., [40, 54, 62]). Thus, individuals with high psychological flexibility can evaluate different solutions to cope with challenging life events and negative experiences more easily. Therefore, psychological flexibility can be a variable that increases the happiness of individuals.

Exposure to psychological maltreatment in childhood may lead to poor psychological flexibility in adulthood. Indeed, Türk et al. [61] found that exposure to psychological maltreatment in childhood negatively predicted psychological flexibility in adulthood. Similarly, low psychological flexibility has been observed in adults exposed to childhood psychological maltreatment [13]. Another study concluded that the presence of childhood psychological maltreatment could lead to a high level of psychological inflexibility in the individual [58]. Low levels of psychological flexibility can harm individuals' subjective happiness. As a matter of fact, Yıldız [72] reported a positive relationship between psychological flexibility and happiness. Other research findings with adults also reveal that decreased psychological flexibility harms psychological resources such as happiness-related life satisfaction and well-being (e.g., [19, 38]. Therefore, psychological flexibility may have a mediating role in the relationship between childhood psychological maltreatment and subjective happiness.

Another variable that may mediate the relationship between childhood psychological maltreatment and subjective happiness, such as psychological flexibility, is family conflict. Family conflict refers to disagreement between family members on various issues [48]. Family conflict can occur between different family members, such as between parents, parent–child, or siblings [2]. Those who were subjected to childhood psychological maltreatment are at high risk of experiencing conflict with family members in the future. The findings of the Tran et al. [59] study support this opinion. The afore-mentioned study reported a positive relationship between childhood psychological maltreatment and exposure to parental conflict. Conflict within the family can be transferred to social environments outside the family over time. In fact, researchers have indicated that children growing up in a conflicted family exhibit antisocial behaviors such as aggression and bullying (e.g., [2, 16]). A conflicting family environment also increases the risk of family members experiencing psychological problems [36, 46]. Additionally, since conflict in the family tends to create negative emotions, high-conflict families may be less happy. Examining related studies, North et al. [50] examined family factors that predict happiness in married individuals over a 10-year period. The study concluded that lower family conflict was linked to higher happiness. Therefore, family conflict is thought to have a mediating role in the relationship between childhood psychological maltreatment and subjective happiness.

Psychological flexibility plays a significant role in shaping individuals' ability to manage interpersonal conflicts, particularly within family relationships [18]. Low psychological flexibility, characterized by cognitive rigidity and emotional avoidance, may hinder effective communication and conflict resolution within the family [17]. In response to difficulties in emotional regulation and adaptive coping, individuals with low psychological flexibility may engage in maladaptive interaction patterns, leading to heightened family conflict. Within this framework, reduced psychological flexibility may increase the risk of family conflict, reinforcing relational distress. Therefore, psychological inflexibility and family conflict are posited to have a serial mediating role in the relationship between childhood psychological maltreatment and subjective happiness.

### The Present Study

Childhood psychological maltreatment is a significant risk factor that negatively affects well-being. However, the mechanisms underlying its impact on subjective happiness remain unclear. Prior research suggests that psychological flexibility, an essential personal trait, may buffer the negative effects of adversity, whereas family conflict, an environmental factor, may intensify them (e.g., [19, 46]). However, the combined role of these factors in the relationship between childhood psychological maltreatment and happiness has not been thoroughly examined. This study aims to address this gap by investigating the serial mediating roles of psychological flexibility and family conflict in the association between childhood psychological maltreatment and subjective happiness. Understanding these mechanisms is crucial for developing targeted interventions that enhance psychological flexibility and reduce family conflict, ultimately improving well-being in individuals with a history of childhood psychological maltreatment. Based on all these, the research questions of this research were determined as follows:

- *RQ1*. Does psychological flexibility mediate the relationship between childhood psychological maltreatment and subjective happiness?
- *RQ2*. Does family conflict mediate the relationship between childhood psychological maltreatment and subjective happiness?
- *RQ3*. Do psychological flexibility and family conflict serially mediate the relationship between childhood psychological maltreatment and subjective happiness?

## Method

### Participant and Procedure

This study, which used convenience sampling methods, involved a total of 493 university students, 304 females (61.7%) and 189 males (38.3%). The participants ranged in age from 18 to 53, and their mean age was 24.02 years (*SD* = 6.342 years). In terms of socioeconomic status, the majority of the sample reported that they perceived themselves at a moderate level (*n* = 303, 61.5%). During the research procedure, ethical permission for the research was first obtained from the National Defense University Social and Human Sciences Ethics Committee (Reference Number = E-35592990-050.04-4010788). Additionally, the ethical rules stated in the Declaration of Helsinki were adhered to throughout the research. Research data were collected online using a structured self-report survey, and participation was entirely voluntary. All participants provided informed consent before beginning the study. Participants were eligible for inclusion if they were at least 18 years old, enrolled in a university program, and had no diagnosed psychiatric disorders. The study did not pay any fees to the participants.

### Measures

#### Psychological Maltreatment Questionnaire (PMQ)

This scale was developed by Arslan [7, 9]. The one-dimensional scale has a total of 12 items (e.g., “He/she would threaten to hurt someone or something I loved”). The higher the possible scores after the reverse items are arranged in the four-point scale, the more the individual is exposed to psychological maltreatment. Items are rated on a four-point scale from 1 (never) to 4 (always). The Cronbach alpha reliability value of the scale was reported as 0.87. In this study, the reliability of the scale was acceptable (ω = 0.86, α = 0.86, λ = 0.88).

#### Acceptance and Action Questionnaire-II (AAQ-II)

Yavuz et al. [71] examined the psychometric properties of the scale developed by Hayes et al. [31] in Turkish culture. This scale, which has seven items (e.g., “I worry about not being able to control my worries and feelings”), is scored on a seven-point scale. Items are rated on a seven-point scale from 1 (It's not suitable) to 7 (completely appropriate). This scale does not contain any reverse-coded items. Possible high scores obtained from the scale indicate psychological rigidity and possible low scores indicate psychological flexibility. This means that for the measurement of psychological flexibility, all items of the scale should be scored after being reversed. The reliability value of the adapted scale was found to be 0.84. In this study, the reliability of the scale was acceptable (ω = 0.91, α = 0.91, λ = 0.91).

#### Family Conflict Scale (FCS)

This scale developed by Akat [2] contains a total of 12 items (e.g., “Family members engage in behaviors that hurt each other”). The scale is scored on a five-point scale and does not contain any reverse items. Items are rated on a five-point scale from 1 (never) to 5 (always). The minimum score that can be obtained from the scale is 12 and the maximum score is 60. Higher scores indicate that individuals experience more family conflict. During the development phase of the scale, Cronbach alpha internal consistency value was reported as 0.93. In this study, the reliability of the scale was acceptable (ω = 0.94, α = 0.94, λ = 0.95).

#### Subjective Happiness Scale (SHS)

This scale, developed by Lyubomirsky and Lepper [47], was adapted to Turkish culture by Akın and Satıcı [3]. The last item of the four-item scale (e.g., “Some people are usually very happy, enjoying everything, no matter what is going on.” To what extent does such a statement describe you?”) is scored reversely. After the reverse item is arranged, a total score is obtained from the scale, which is scored in a seven-point rating form. Items are rated on a seven-point scale from 1 (It's not suitable) to 7 (completely appropriate). As the scores that can be obtained from the scale increase, the subjective happiness level of individuals increases. In the adaptation study, the Cronbach alpha value of the scale was reported as 0.86. In this study, the reliability of the scale was acceptable (ω = 0.75, α = 0.73, λ = 0.71).

### Data Analysis

In this study, firstly the normality test of the data was examined. After ensuring normal distribution, descriptive statistics, correlation coefficients, and different reliability values (Cronbach alpha, McDonald omega, and Guttman lambda) were calculated. During the data cleaning process, 23 outliers were identified and removed to ensure the accuracy and reliability of the analyses. Then, two-stage structural equation modeling suggested by Anderson and Gerbing [6] was conducted to test the research questions of the study. The first of these two stages is to examine the measurement model, and the second is to test the structural model. In both models, the fit index values are examined to determine the fit between the model and the data. The literature states that the values of the Comparative Fit Index (CFI), Tucker-Lewis Index (TLI), Normed Fit Index (NFI), Goodness-of-Fit Index (GFI), and Incremental Fit Index (IFI) should exceed 0.90, while the values of the Standardized Root Mean Square Residual (SRMR) and Root Mean Square Error of Approximation (RMSEA) should be less than 0.08 [35]. Lastly, gender and age were included in the model as control variables. These calculations were performed using SPSS 26.0, JASP 0.11.1, AMOS Graphics 24.0 statistical package programs.

## Results

### Preliminary Analyses

The findings from the preliminary analyses are presented in Table 1. First, normality assumptions were tested, followed by the calculation of descriptive statistics and reliability coefficients for the study variables. Additionally, a correlation analysis was conducted to examine the relationships between variables. The results indicated that all variables were normally distributed and demonstrated sufficient reliability. Furthermore, all variables were significantly correlated with each other. Childhood psychological maltreatment was negatively correlated with psychological flexibility (*r* =  − 0.41, *p* < 0.01) and subjective happiness (*r* =  − 0.34, *p* < 0.01), while it was positively correlated with family conflict (*r* = 0.55, *p* < 0.01). Additionally, psychological flexibility was negatively associated with family conflict (*r* =  − 0.45, *p* < 0.01) and positively associated with subjective happiness (*r* = 0.50, *p* < 0.01). Lastly, a significant negative correlation was observed between family conflict and subjective happiness (*r* =  − 0.25, *p* < 0.01).

**Table 1:** Descriptive statistics, reliabilities, normality assumptions, and correlations for the study variables

Variable	1	2	3	4
1. *Childhood psychological maltreatment*	–			
2. *Psychological flexibility*	−0.411^**^	–		
3. *Family conflict*	0.549^**^	−0.447^**^	–	
4. *Subjective happiness*	−0.336^**^	0.496^**^	−0.249^**^	–
Mean	19.440	31.594	33.107	17.572
SD	5.359	10.481	11.264	4.626
Skewness	1.067	−0.307	−0.004	−0.366
Kurtosis	1.705	−0.576	−0.604	−0.088
McDonald’s ω	0.868	0.918	0.944	0.753
Cronbach’s α	0.861	0.917	0.944	0.732
Guttman’s λ6	0.886	0.919	0.950	0.717

^**^p < 0.01

### Structural Equation Modeling

Following the completion of the preliminary analyses, a two-stage structural equation modeling (SEM) analysis was conducted using the AMOS Graphics statistical package program. Then, the significance of the mediating roles was tested using the bootstrapping method. In the first stage, the measurement model included four latent constructs (childhood psychological maltreatment, psychological flexibility, family conflict, and subjective happiness) and 12 observed variables. The fit indices indicated an acceptable model fit: *χ*^*2*^_(*48, N* = *493*)_ = 203.895, *p* < 0.001; *χ*^*2*^*/df* = 4.25; CFI = 0.961; TLI = 0.946; NFI = 0.950; GFI = 0.938; IFI = 0.961; RMSEA = 0.080. Moreover, all factor loadings ranged from 0.33 to 0.97 and were statistically significant. Although the RMSEA value (0.080) is at the borderline of acceptability, the overall model fit indices (CFI, TLI, NFI, GFI, IFI) suggest an acceptable measurement model fit. Given that RMSEA is sensitive to sample size and model complexity, and considering the strong support from other fit indices, the measurement model can be considered robust. This means that the observed variables adequately manipulate the latent variables.

**Fig. 1:**
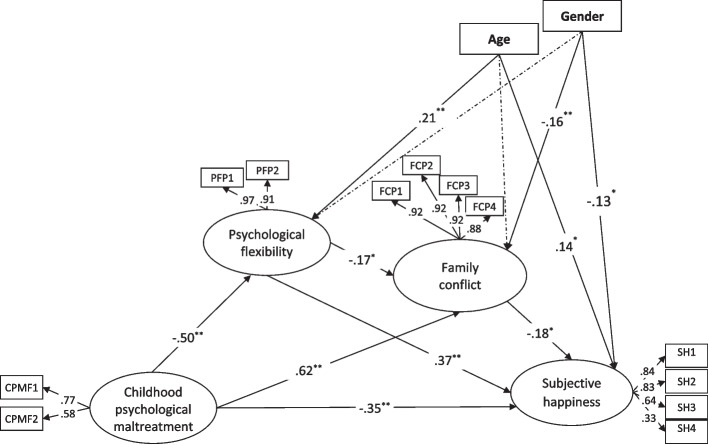
The serial mediation model showing childhood psychological maltreatment, psychological flexibility, family conflict on subjective happiness, ^*^ p < 0.05 ^**^ p < 0.001. *Note.* Values shown are standardized coefficients. Non-significant pathways are presented with dotted lines. CPMF = factors of childhood psychological maltreatment; PFP = parcels of psychological flexibility; FCP = parcels of family conflict; SH = items of subjective happiness

Following the measurement model, the structural model was tested. Gender and age were included as control variables in this model. Controlling for these variables helps ensure that the observed relationships among childhood psychological maltreatment, psychological flexibility, family conflict, and subjective happiness are not confounded by demographic differences. The analysis results indicated that all paths in the structural model were statistically significant, and the fit indices demonstrated an acceptable model fit: *χ*^*2*^_(*66, N* = *493*)_ = 251.301, *p* < 0.001; *χ*^*2*^*/df* = 3.808; CFI = 0.955; TLI = 0.938; NFI = 0.940; GFI = 0.934; IFI = 0.955; RMSEA = 0.076 (see Fig. 1). This finding suggests that psychological flexibility and family conflict serially mediate the relationship between childhood psychological maltreatment and subjective happiness.

The significance of the mediating roles obtained was tested using the bootstrapping method. Table 2 presents the standardized coefficients for the indirect effects of the variables. As shown in Table 2, childhood psychological maltreatment was found to significantly predict subjective happiness through psychological flexibility (*B* =  − 0.15, 95% CI [− 0.24, − 0.09]). Furthermore, childhood psychological maltreatment significantly predicted subjective happiness via family conflict (*B* = 0.09, 95% CI [0.01, 0.23]). Addressing the main research questions of the study, it was revealed that childhood psychological maltreatment predicted subjective happiness through psychological flexibility and family conflict (*B* = 0.01, 95% CI [0.001, 0.035]). These findings suggest that psychological flexibility and family conflict play a serial and partial mediating role in the relationship between childhood psychological maltreatment and subjective happiness.

**Table 2:** Indirect effect of serial mediation model

		95%CI	
Path	Coefficient	*LL*	*UL*
CPM →Psychological flexibility → Subjective happiness	−0.149	−0.235	−0.090
CPM → Family conflict → Subjective happiness	0.090	0.008	0.229
CPM → Psychological flexibility → Family conflict → Subjective happiness	0.012	0.001	0.035

*CPM* childhood psychological maltreatment; *CI* confidence interval; *LL* lower limit; *UL* upper limit

## Discussion

Childhood experiences are one of the most effective factors in terms of happiness experiences in adulthood. Negative experiences during childhood can have long-term negative effects on individuals and adversely influence their level of happiness during adulthood. Moreover, as stated prior, the interaction of individual and environmental factors is associated with happiness. In this study, the serial mediating roles of psychological flexibility and family conflict in the relationship between childhood psychological maltreatment and subjective happiness were examined in order to reveal individual and environmental factors affecting happiness. The findings of our study revealed that both psychological flexibility and family conflict played a serial mediating role in the relationship between childhood psychological maltreatment and subjective happiness. The findings of the study are discussed in detail below.

The first finding of the study is that psychological flexibility mediates the relationship between childhood psychological maltreatment and subjective happiness (*RQ1*). In other words, the first finding of the study revealed that exposure to maltreatment in childhood reduces psychological flexibility and that the decrease in psychological flexibility negatively influences the subjective happiness of individuals. This finding of our study is similar to previous research showing that childhood maltreatment harms psychological flexibility. Indeed, Türk et al. [61] concluded that childhood psychological maltreatment adversely affects psychological flexibility. Taşören [58] also reported that childhood maltreatment increases the psychological inflexibility levels of individuals. Similarly, Boykin et al. [14] concluded that childhood psychological maltreatment and psychological inflexibility were high. In other words, they mentioned that childhood psychological maltreatment reduces psychological flexibility. Baugh et al. [13] also reported a negative relationship between childhood psychological maltreatment and psychological flexibility. The reason for the finding in our study that childhood psychological maltreatment is negatively associated with psychological flexibility may be that individuals who were abused in childhood develop inflexible cognitions about the world and other people being bad.

Low psychological flexibility may be associated with lower levels of happiness. Since happiness consists of an individual's evaluations of his or her life, psychological flexibility is an important factor. As a matter of fact, Demirci-Seyrek & Ersanlı [21] also revealed that those with high psychological flexibility feel happy, while those with low psychological flexibility feel unhappy. Similarly, Howell and Demuynck [34] concluded that psychological flexibility increases the positive influence level of individuals. Another recent study reported that those with low psychological flexibility also have low happiness levels [72]. As a result, exposure to childhood psychological maltreatment reduces the psychological flexibility levels of individuals. Having low psychological flexibility harms happiness because it makes it difficult to adapt to different life events and accept negative experiences and emotions. These findings highlight the cognitive consequences of childhood psychological maltreatment and suggest that interventions aimed at enhancing psychological flexibility may help mitigate its negative effects on well-being.

The second finding of the study is that family conflict mediates the relationship between childhood psychological maltreatment and subjective happiness (*RQ2*). In other words, according to the second finding of our research, being exposed to childhood psychological maltreatment is linked to an individual experiencing more conflict with family members, which in turn predicts lower subjective happiness. Exposure to childhood psychological maltreatment may lead individuals to develop irrational thoughts in response to positive emotions such as happiness. For instance, those who are subjected to psychological maltreatment may think that they may be punished or that something bad may happen to them after feeling happy [56]. Therefore, these individuals may develop a fear of happiness, characterized by avoiding it or approaching it with caution. Indeed, Arslan [10] obtained a positive relationship between childhood psychological maltreatment and fear of happiness.

Being exposed to childhood psychological maltreatment can lead to conflict in social relationships, especially in the family. Family conflict can cause family members to experience emotional problems [51]. In addition, numerous studies have revealed that family conflict is a risk factor for psychological problems (e.g., [26, 57]). Considering that psychological problems negatively influence individuals' happiness, it can be said that family conflict reduces the level of happiness. Other studies have also observed similar results. For instance, North et al. [50] found that lower family conflict was associated with greater happiness. Recent studies suggest that work-family conflict reduces happiness levels (e.g., [28, 53]). In conclusion, childhood psychological maltreatment leads to conflict in the family, and a conflicting family environment harms the happiness of individuals. These results emphasize the role of family conflict as a critical mediator between childhood adversity and well-being, underscoring the need for family-based interventions to mitigate its negative effects on happiness.

Lastly, this study found that psychological flexibility and family conflict had a significant serial mediating role in the relationship between childhood psychological maltreatment and subjective happiness (*RQ3*). This finding supports the idea that individuals with low psychological flexibility due to exposure to childhood psychological maltreatment will experience more conflict with family members, which will reduce their happiness levels. This finding is consistent with previous studies showing that childhood psychological maltreatment damages the cognitive characteristics of individuals (e.g., [1, 37]). Additionally, it is consistent with previous research findings that people with low psychological flexibility experience problems in family relationships [18, 27], which in turn reduces their happiness [25, 53]. This study has revealed that exposure to childhood psychological maltreatment is an important risk factor for individuals' subjective happiness. Furthermore, the study has determined that psychological flexibility plays a crucial role in preserving individuals' happiness. Finally, research has demonstrated that childhood exposure to negative experiences increases family conflict, which negatively impacts individuals' happiness. These findings underscore the interconnected nature of psychological flexibility and family dynamics in shaping well-being, highlighting the importance of targeted interventions that strengthen psychological resilience and reduce family conflict to enhance happiness in individuals affected by childhood adversity.

### Limitations and Future Directions

Although this study makes significant contributions to the relevant literature, it has some limitations. Firstly, the cross-sectional design of this study precluded the establishment of a cause-effect relationship. Since childhood psychological maltreatment and its effects on subjective happiness are dynamic processes that unfold over time, a longitudinal or experimental research design would provide a more comprehensive understanding of these relationships. Future studies should employ such designs to determine the causal links between childhood psychological maltreatment and subjective happiness. Secondly, despite conducting the study on adults in alignment with its purpose, the sample group presents some limitations. The psychological effects of childhood maltreatment may differ across developmental stages, as coping mechanisms and resilience factors evolve over time. In future studies, examining these variables in diverse age groups (such as adolescents, young adults, and the elderly) will enhance the generalizability of the findings. Thirdly, the use of convenience sampling methods in this study also hinders the generalizability of the findings. Participants recruited through convenience sampling may not represent the broader population, which could lead to sampling bias. Therefore, it is recommended to use different sampling methods in future studies. Additionally, the generalizability of the findings may be limited by cultural factors, as psychological maltreatment and its effects on happiness can vary across cultures. Moreover, as the study relies on self-report measures from a single data source, potential biases such as common method bias should be considered. Finally, in this study, one of the mediating variables in the relationship between childhood psychological maltreatment and subjective happiness is psychological flexibility. However, emotional variables may also play a significant mediating role in this relationship. Therefore, future studies can examine emotional characteristics like emotion regulation skills, emotional expression skills, and anger coping as mediating variables. Examining these factors could provide a more comprehensive understanding of the mechanisms underlying the relationship between childhood psychological maltreatment and happiness.

### Implications

The findings of this study provide important insights for both mental health professionals and researchers. Firstly, the study found that psychological flexibility plays a mediating role between childhood psychological maltreatment and subjective happiness. In other words, psychological flexibility has been shown to prevent the long-term effects of childhood psychological maltreatment from decreasing happiness in individuals. Therefore, mental health professionals should incorporate interventions that enhance psychological flexibility in therapy and counseling sessions. Individual or group-based psychological flexibility training, particularly within the framework of Acceptance and Commitment Therapy (ACT), could help individuals exposed to childhood maltreatment develop adaptive coping strategies.

Secondly, this study found that family conflict significantly mediates the relationship between childhood psychological maltreatment and subjective happiness. This highlights the importance of addressing family conflict in therapeutic settings. Mental health professionals should implement interventions aimed at improving family communication, conflict resolution, and emotional regulation. In addition, structured family therapy approaches, such as systemic family therapy, could be effective in mitigating family conflict and fostering healthier family dynamics. Lastly, this study concluded that psychological flexibility and family conflict together had significant serial mediating roles in the relationship between childhood psychological maltreatment and subjective happiness. These findings suggest that a comprehensive intervention approach targeting both individual (psychological flexibility) and environmental (family conflict) factors could be more effective in promoting well-being. Future research should further explore these mechanisms using longitudinal designs and diverse populations to better understand their long-term effects.

## Conclusion

This study underscores the potential influence of psychological flexibility and family conflict in mediating the relationship between childhood psychological maltreatment and subjective happiness. The findings indicate that promoting psychological flexibility and strengthening conflict resolution skills may play a crucial role in alleviating the adverse impact of childhood psychological maltreatment on overall well-being. Targeted interventions addressing these factors could help enhance subjective happiness among individuals who have experienced such adverse childhood conditions.

## Data Availability

Data will be available on request.

## Declarations

### Pre-registration Statement

This study was not pre-registered.

## References

[1] Ainamani HE, Rukundo GZ, Nduhukire T, Ndyareba E, Hecker T. Child maltreatment, cognitive functions and the mediating role of mental health problems among maltreated children and adolescents in Uganda. Child Adolesc Psychiatry Ment Health. 2021;15(22):1–11. 10.1186/s13034-021-00373-7. 33941232 10.1186/s13034-021-00373-7 PMC8091686

[2] Akat M. Intergenerational transmission of family conflict and trait anxity: serial mediation of adolescent positive youth development and aggression. [Unpublished Doctoral Thesis]. Necmettin Erbakan University 2024.

[3] Akın A, Satıcı SA. Subjective happiness scale: a study of validity and reliability. Sakarya Univer J Educ Fac. 2011;21(21):65–77.

[4] Allen B. Childhood psychological abuse and adult aggression: The mediating role of self-capacities. J Interpers Violence. 2011;26(10):2093–110. 10.1177/0886260510383035. 20956437 10.1177/0886260510383035

[5] American Professional Society on the Abuse of Children (2019). *The Investigation and determination of suspected psychological maltreatment in children and adolescents*. https://www.apsac.org/guidelines. Accessed 11 October 2024.

[6] Anderson JC, Gerbing DW. Structural equation modeling in practice: a review and recommended two-step approach. Psychol Bull. 1988;103(3):411–23. 10.1037/0033-2909.103.3.411.

[7] Arslan G. Development psychological maltreatment questionnaire (PMQ): investigating psychometric properties in adolescents. Bartın Univer J Fac Educ. 2015;4(2):727–38. 10.14686/buefad.v4i2.5000146983.

[8] Arslan G. Psychological maltreatment, emotional and behavioral problems in adolescents: the mediating role of resilience and self-esteem. Child Abuse Negl. 2016;52:200–9. 10.1016/j.chiabu.2015.09.010. 26518981 10.1016/j.chiabu.2015.09.010

[9] Arslan G. Psychological maltreatment, coping strategies, and mental health problems: a brief and effective measure of psychological maltreatment in adolescents. Child Abuse Negl. 2017;68:96–106. 10.1016/j.chiabu.2017.03.023. 28427000 10.1016/j.chiabu.2017.03.023

[10] Arslan G. Childhood psychological maltreatment, optimism, aversion to happiness, and psychological adjustment among college students. Curr Psychol. 2023;42:25142–50. 10.1007/s12144-022-03538-5. 10.1007/s12144-022-03538-5 PMC9379216 35990208

[11] Arslan G. Psychological maltreatment and substance use among college students: psychological distress, belongingness, and social support. J Ethn Subst Abuse. 2024;23(3):426–49. 10.1080/15332640.2022.2122098. 36129727 10.1080/15332640.2022.2122098

[12] Arslan G, Genç E, Yıldırım M, Tanhan A, Allen KA. Psychological maltreatment, meaning in life, emotions, and psychological health in young adults: a multi-mediation approach. Child Youth Serv Rev. 2022;132:106296. 10.1016/j.childyouth.2021.106296.

[13] Baugh LM, Cox DW, Young RA, Kealy D. Partner trust and childhood emotional maltreatment: the mediating and moderating roles of maladaptive schemas and psychological flexibility. J Contextual Behav Sci. 2019;12:66–73. 10.1016/j.jcbs.2019.02.001.

[14] Boykin DM, Himmerich SJ, Pinciotti CM, Miller LM, Miron LR, Orcutt HK. Barriers to self-compassion for female survivors of childhood maltreatment: the roles of fear of self-compassion and psychological inflexibility. Child Abuse Negl. 2018;76:216–24. 10.1016/j.chiabu.2017.11.003. 29144981 10.1016/j.chiabu.2017.11.003

[15] Bowlby J. *A secure base: Parent-child attachment and healthy human development*. New York: Basic Books; 1988.

[16] Chen J-K, Yang B, Lin C-Y, Wang L-C. Affiliation with delinquent peers as a mediator of the relationships between family conflict and school bullying: a short-term longitudinal panel study. J Interpers Violence. 2023;38(19–20):10686–702. 10.1177/08862605231175517. 37226726 10.1177/08862605231175517

[17] Daks JS, Peltz JS, Rogge RD. Psychological flexibility and inflexibility as sources of resiliency and risk during a pandemic: modeling the cascade of COVID-19 stress on family systems with a contextual behavioral science lens. J Contextual Behav Sci. 2020;18:16–27. 10.1016/j.jcbs.2020.08.003. 32834972 10.1016/j.jcbs.2020.08.003 PMC7428754

[18] Daks JS, Peltz JS, Rogge RD. The impact of psychological flexibility on family dynamics amidst the COVID-19 pandemic: a longitudinal perspective. J Contextual Behav Sci. 2022;26:97–113. 10.1016/j.jcbs.2022.08.011. 36105870 10.1016/j.jcbs.2022.08.011 PMC9461241

[19] Dawson DL, Golijani-Moghaddam N. COVID-19: psychological flexibility, coping, mental health, and wellbeing in the UK during the pandemic. J Contextual Behav Sci. 2020;17:126–34. 10.1016/j.jcbs.2020.07.010. 32834970 10.1016/j.jcbs.2020.07.010 PMC7392106

[20] Deci EL, Ryan RM. The “what” and “why” of goal pursuits: Human needs and the self-determination of behavior. Psychol Inq. 2000;11(4):227–68. 10.1207/S15327965PLI1104_01.

[21] Demirci Seyrek Ö, Ersanlı K. The relationship between meaning in life and psychologıcal flexibility of university students. Turk Stud. 2017;12(4):143–62. 10.7827/TurkishStudies.10053.

[22] Dfarhud D, Malmir M, Khanahmadi M. Happiness & health: the biological factors- systematic review article. Iran J Public Health. 2014;43(11):1468–77. 26060713 PMC4449495

[23] Diener E. Subjective well-being. Psychol Bull. 1984;95(3):542–75. 10.1037/0033-2909.95.3.542. 6399758

[24] Diener E, Scollon CN, Lucas RE. The evolving concept of subjective well-being: The multifaceted nature of happiness. In E. Diener (Ed.), *Assessing well-being* (pp. 67–100). Springer. 2009. 10.1007/978-90-481-2354-4_4

[25] El Keshky MES, Sarour EO. The relationships between work-family conflict and life satisfaction and happiness among nurses: a moderated mediation model of gratitude and self-compassion. Front Public Health. 2024;12:1340074. 10.3389/fpubh.2024.1340074. 38450130 10.3389/fpubh.2024.1340074 PMC10914949

[26] Flores SM, Salum GA, Manfro GG. Dysfunctional family environments and childhood psychopathology: the role of psychiatric comorbidity. Trends Psychiatry Psychother. 2014;36(3):147–51. 10.1590/2237-6089-2014-0003. 27003846 10.1590/2237-6089-2014-0003

[27] Flujas-Contreras JM, Recio-Berlanga A, Andrés MP, Fernández-Torres M, Sánchez-López P, Gómez I. The relationship between parental stress and psychological adjustment of the children: the role of parental psychological flexibility as a mediator. J Contextual Behav Sci. 2023;29:202–8. 10.1016/j.jcbs.2023.07.006.

[28] Gümüş Dönmez F, Gürlek M, Karatepe OM. Does work-family conflict mediate the effect of psychological resilience on tour guides’ happiness? Int J Contemp Hosp Manag. 2023;36(9):2932–54. 10.1108/IJCHM-01-2023-0077.

[29] Hart SN, Brassard MR, Binggeli NJ, Davidson HA. Psychological maltreatment. In: Myers JEB, Berliner L, Briere J, Hendrix CT, Jenny C, Reid T, editors. The APSAC handbook on child maltreatment. 2nd ed. Thousand Oaks: Sage Publications; 2002. pp. 79–103.

[30] Hayes SC, Levin ME, Plumb-Vilardaga J, Villatte JL, Pistorello J. Acceptance and commitment therapy and contextual behavioral science: examining the progress of a distinctive model of behavioral and cognitive therapy. Behav Ther. 2013;44(2):180–98. 10.1016/j.beth.2009.08.002. 23611068 10.1016/j.beth.2009.08.002 PMC3635495

[31] Hayes SC, Strosahl KD, Wilson KG, Bissett RT, Pistorello J, Toarmino D, Polusny MA, Dykstra TA, Batten SV, Bergan J, Stewart SH, Zvolensky MJ, Eifert GH, Bond FW, Forsyth JP, Karekla M, McCurry SM. Measuring experiential avoidance: a preliminary test of a working model. Psychol Rec. 2004;54(4):553–78. 10.1007/BF03395492.

[32] Hayes SC, Wilson KG, Gifford EV, Follette VM, Strosahl K. Experiential avoidance and behavioral disorders: a functional dimensional approach to diagnosis and treatment. J Consult Clin Psychol. 1996;64(6):1152–68. 10.1037/0022-006X.64.6.1152. 8991302 10.1037//0022-006x.64.6.1152

[33] Herrenkohl TI, Klika JB, Herrenkohl RC, Russo MJ, Dee T. A prospective investigation of the relationship between child maltreatment and indicators of adult psychological well-being. Violence Vict. 2012;27(5):764–76. 10.1891/0886-6708.27.5.764. 23155725 10.1891/0886-6708.27.5.764 PMC3501987

[34] Howell AJ, Demuynck KM. Psychological flexibility and psychological inflexibility are independently associated with both hedonic and eudaimonic well-being. J Contextual Behav Sci. 2021;20:163–71. 10.1016/j.jcbs.2021.04.002.

[35] Hu L-T, Bentler PM. Cutoff criteria for fit indexes in covariance structure analysis: conventional criteria versus new alternatives. Struct Equ Model. 1999;6(1):1–55. 10.1080/10705519909540118.

[36] Juang LP, Syed M, Takagi M. Intergenerational discrepancies of parental control among Chinese American families: links to family conflict and adolescent depressive symptoms. J Adolesc. 2007;30(6):965–75. 10.1016/j.adolescence.2007.01.004. 17360033 10.1016/j.adolescence.2007.01.004

[37] Kalia V, Knauft K, Hayatbini N. Cognitive flexibility and perceived threat from COVID-19 mediate the relationship between childhood maltreatment and state anxiety. PLoS ONE. 2020;15(12):e0243881. 10.1371/journal.pone.0243881. 33306748 10.1371/journal.pone.0243881 PMC7732062

[38] Karakuş S, EvinAkbaş S. The mediating role of psychological flexibility in explaining authenticity and life satisfaction with alexithymia. Participatory Educ Res. 2022;9(1):285–302. 10.17275/per.22.16.9.1.

[39] Kashdan TB, Rottenberg J. Psychological flexibility as a fundamental aspect of health. Clin Psychol Rev. 2010;30:865–878. 10.1016/j.cpr.2010.03.001. 21151705 10.1016/j.cpr.2010.03.001 PMC2998793

[40] Kaya EM, Eken OF, Ümmet D. The predictor effect of insight and cognitive flexibility on psychological hardiness. Cyprus Turk J Psychiatry Psychol. 2021;3(1):22–9. 10.35365/ctjpp.21.1.05.

[41] Kim-Spoon J, Cicchetti D, Rogosch FA. A longitudinal study of emotion regulation, emotion lability-negativity, and internalizing symptomatology in maltreated and nonmaltreated children. Child Dev. 2012;84(2):512–27. 10.1111/j.1467-8624.2012.01857.x. 23034132 10.1111/j.1467-8624.2012.01857.x PMC3794707

[42] Konca AS. Digital technology usage of young children: screen time and families. Early Childhood Educ J. 2022;50(7):1097–108. 10.1007/s10643-021-01245-7.

[43] Lee H, Kim E. Global prevalence of physical and psychological child abuse during COVID-19: a systematic review and meta-analysis. Child Abuse Negl. 2023;135:105984. 10.1016/j.chiabu.2022.105984. 36538870 10.1016/j.chiabu.2022.105984 PMC9722678

[44] Levin ME, Hildebrandt MJ, Lillis J, Hayes SC. The impact of treatment components suggested by the psychological flexibility model: a meta-analysis of laboratory-based component studies. Behavior Ther. 2012;43:741–756. 10.1016/j.beth.2012.05.003. 10.1016/j.beth.2012.05.003 23046777

[45] Liu F, Zhang Z, Chen L. Mediating effect of neuroticism and negative coping style in relation to childhood psychological maltreatment and smartphone addiction among college students in China. Child Abuse Negl. 2020;106:104531. 10.1016/j.chiabu.2020.104531. 32447143 10.1016/j.chiabu.2020.104531

[46] Low YTA. Family conflicts, anxiety and depressive symptoms, and suicidal ideation of Chinese adolescents in Hong Kong. Appl Res Qual Life. 2021;16:2457–73. 10.1007/s11482-021-09925-7.

[47] Lyubomirsky S, Lepper HS. A measure of subjective happiness: preliminary reliability and construct validation. Soc Indic Res. 1999;46(2):137–55. 10.1023/A:1006824100041.

[48] Marta E, & Alfieri S. Family Conflicts. In Michalos, A. C. (Eds.). *Encyclopedia of quality of life and well-being research.* Springer. 2014. 10.1007/978-94-007-0753-5_997

[49] Moran DJ. Acceptance and commitment training in the workplace. Curr Opin Psychol. 2015;2:26–31. 10.1016/j.copsyc.2014.12.031.

[50] North RJ, Holahan CJ, Moos RH, Cronkite RC. Family support, family income, and happiness: a 10-year perspective. J Fam Psychol. 2008;22(3):475–83. 10.1037/0893-3200.22.3.475. 18540776 10.1037/0893-3200.22.3.475

[51] Ogan MA, Monk JK, Thibodeau-Nielsen RB, Vennum A, Soloski K. The role of emotional dysregulation in the association between family-of-origin conflict and romantic relationship maintenance. J Marital Fam Ther. 2024;50(1):28–44. 10.1111/jmft.12667. 37752739 10.1111/jmft.12667

[52] Ong CW, Sheehan KG, Haaga DAF. Measuring ACT in context: challenges and future directions. J Contextual Behav Sci. 2023;28:235–47. 10.1016/j.jcbs.2023.04.005.

[53] Pan Y, Aisihaer N, Li Q, Jiao Y, Ren S. Work-family conflict, happiness and organizational citizenship behavior among professional women: a moderated mediation model. Front Psychol. 2022;13:923288. 10.3389/fpsyg.2022.923288. 35774962 10.3389/fpsyg.2022.923288 PMC9237458

[54] Pyszkowska A, Rönnlund M. Psychological flexibility and self-compassion as predictors of well-being: mediating role of a balanced time perspective. Front Psychol. 2021;12:671746. 10.3389/fpsyg.2021.671746. 34177730 10.3389/fpsyg.2021.671746 PMC8222535

[55] Sağar M. Psychologıcal flexibility and problem solving skills as predictors of social media addiction in adults. Mehmet Akif Ersoy Univer J Soc Sci Inst. 2022;35:179–92. 10.20875/makusobed.1080674.

[56] Satıcı SA, Yılmaz FB, Karaağaç ZG, Okur S. From childhood psychological maltreatment to fear of happiness: exploring the serial mediation of external shame and family communication. Child Youth Serv Rev. 2024;157:107425. 10.1016/j.childyouth.2023.107425.

[57] Sela Y, Zach M, Amichay-Hamburger Y, Mishali M, Omer H. Family environment and problematic internet use among adolescents: the mediating roles of depression and fear of missing out. Comput Hum Behav. 2020;106:106226. 10.1016/j.chb.2019.106226.

[58] Taşören AB. Childhood maltreatment and emotional distress: the role of beliefs about emotion and psychological inflexibility. Curr Psychol. 2023;42:13276–87. 10.1007/s12144-021-02594-7. 10.1007/s12144-021-02594-7 PMC8754519 35039733

[59] Tran NK, van Berkel SR, van IJzendoorn MH, Alink LRA. Child and family factors associated with child maltreatment in Vietnam. J Interpersonal Violence. 2021;36(5–6):NP2931–53. 10.1177/0886260518767914. 10.1177/0886260518767914 PMC7941509 29658819

[60] Tsai JL, Louie JY, Chen EE, Uchida Y. Learning what feelings to desire: socialization of ideal affect through children’s storybooks. Pers Soc Psychol Bull. 2007;33(1):17–30. 10.1177/0146167206292749. 17178927 10.1177/0146167206292749

[61] Türk N, Arslan G, Kaya A, Güç E, Turan ME. Psychological maltreatment, meaning-centered coping, psychological flexibility, and suicide cognitions: a moderated mediation model. Child Abuse Negl. 2024;152:106735. 10.1016/j.chiabu.2024.106735. 38569454 10.1016/j.chiabu.2024.106735

[62] Twiselton K, Stanton SCE, Gillanders D, Bottomley E. Exploring the links between psychological flexibility, individual well-being, and relationship quality. Pers Relat. 2020;27(4):880–906. 10.1111/pere.12344.

[63] UNİCEF. Nearly 400 million young children worldwide regularly experience violent discipline at home – UNICEF. 2024. https://www.unicef.org/press-releases/nearly-400-million-young-children-worldwide-regularly-experience-violent-discipline#:~:text=NEW%20YORK%2C%2011%20June%202024,are%20punished%20by%20physical%20means Accessed 27 September 2024.

[64] Wang ZJ, Liu CY, Wang YM, Wang Y. Childhood psychological maltreatment and adolescent depressive symptoms: exploring the role of social anxiety and maladaptive emotion regulation strategies. J Affect Disord. 2024;344:365–72. 10.1016/j.jad.2023.10.046. 37832734 10.1016/j.jad.2023.10.046

[65] Wang Q, Tu R, Hu W, Luo X, Zhao F. Childhood psychological maltreatment and depression among Chinese adolescents: multiple mediating roles of perceived ostracism and core self-evaluation. Int J Environ Res Public Health. 2021;18(21):11283. 10.3390/ijerph182111283. 34769803 10.3390/ijerph182111283 PMC8583377

[66] Wei PC, Yu HQ. The relationship between childhood psychological abuse and social media addiction among college students: the mediating role of fear of missing out and the moderating role of left-behind experience. Arch Med Sci. 2024;20(3):798–805. 10.5114/aoms/174649. 39050150 10.5114/aoms/174649 PMC11264144

[67] World Health Organization. *Child maltreatment*. 2024. https://www.who.int/news-room/fact-sheets/detail/child-maltreatment

[68] World Health Organization. *Investing in children: the European child maltreatment prevention action plan 2015–2020.* 2015. https://iris.who.int/handle/10665/350142 Accessed 5 October 2024

[69] Wu YQ, Liu F, Chan KQ, Wang NX, Zhao S, Sun X, Shen W, Wang ZJ. Childhood psychological maltreatment and internet gaming addiction in Chinese adolescents: mediation roles of maladaptive emotion regulation strategies and psychosocial problems. Child Abuse Negl. 2022;129:105669. 10.1016/j.chiabu.2022.105669. 35598385 10.1016/j.chiabu.2022.105669

[70] Veenhoven R. *Conditions of happiness*. Dordrecht: Publishing Company; 1984.

[71] Yavuz F, Ulusoy S, Iskin M, Esen FB, Burhan HS, Karadere ME, Yavuz N. Turkish version of acceptance and action questionnaire-II (AAQ-II): a reliability and validity analysis in clinical and non-clinical samples. Bull Clin Psychopharmacol. 2016;26(4):397–408. 10.5455/bcp.20160223124107.

[72] Yıldız M. *Happiness, hope, psychological flexibility, acceptance and commitment therapy, high school students, adolescence.* [Unpublished Master's Thesis]. Hasan Kalyoncu University, Gaziantep; 2022.

[73] Yılmaz FB, Satıcı SA. Childhood maltreatment and spiritual well-being: intolerance of uncertainty and emotion regulation as mediators in Turkish sample. J Relig Health. 2024;63:2380–96. 10.1007/s10943-023-01965-7. 38070045 10.1007/s10943-023-01965-7

[74] Zhang Y, Xu W, McDonnell D, Wang JL. The relationship between childhood maltreatment subtypes and adolescent internalizing problems: the mediating role of maladaptive cognitive emotion regulation strategies. Child Abuse Negl. 2024;152:106796. 10.1016/j.chiabu.2024.106796. 38631188 10.1016/j.chiabu.2024.106796

[75] Zhao J, Sun R, Shangguan M. Childhood psychological maltreatment and social anxiety in college students: the roles of parasympathetic nervous system activity and parent-child separation experience. Child Abuse Negl. 2024;151:106723. 10.1016/j.chiabu.2024.106723. 38461709 10.1016/j.chiabu.2024.106723

